# Supplementary material for: Adiabatic elimination-based coupling control in densely packed subwavelength waveguides
Source: Nat Commun. 2015 Jun 26;6:7565. doi: 10.1038/ncomms8565 (PMC4491806; doi:10.1038/ncomms8565)
Supplement: Supplementary Information — Supplementary Figures 1-11, Supplementary Table 1, Supplementary Discussion 1-8, Supplementary Methods 1-2 and Supplementary References. [file ncomms8565-s1.pdf]

Supplementary Information

Active coupling control in densely packed subwavelength waveguides via dark mode interaction

Supplementary Figures

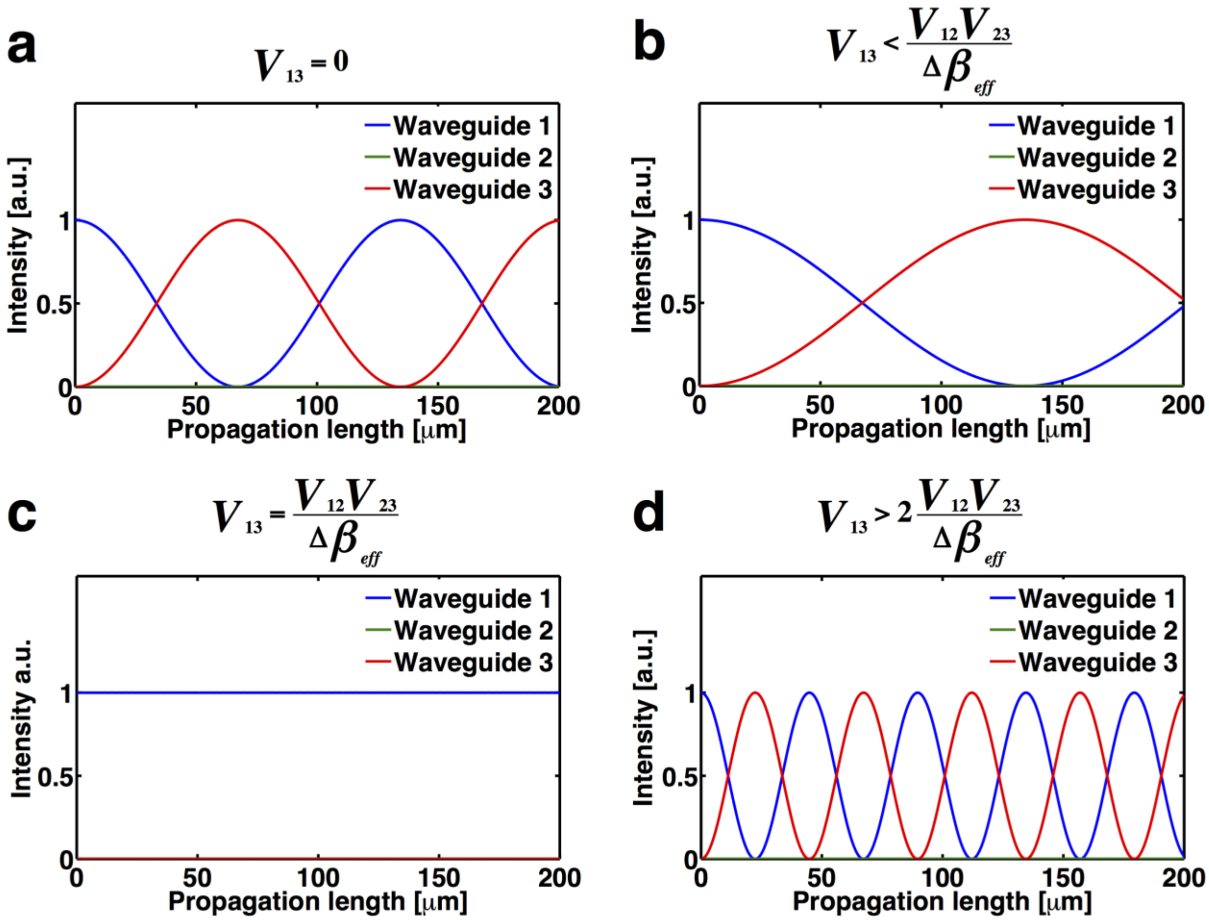

Supplementary Figure 1- Effective coupling in three waveguides with adiabatic elimination approximation and direct 1-3 coupling (a-d) Examples of the dynamical evolution for several AE configurations with different relations between the direct coupling parameter $\boldsymbol{V_{13}}$ and the indirect coupling $\frac{\boldsymbol{V_{12}V_{23}}}{\boldsymbol{\Delta\beta_{23}}}$.

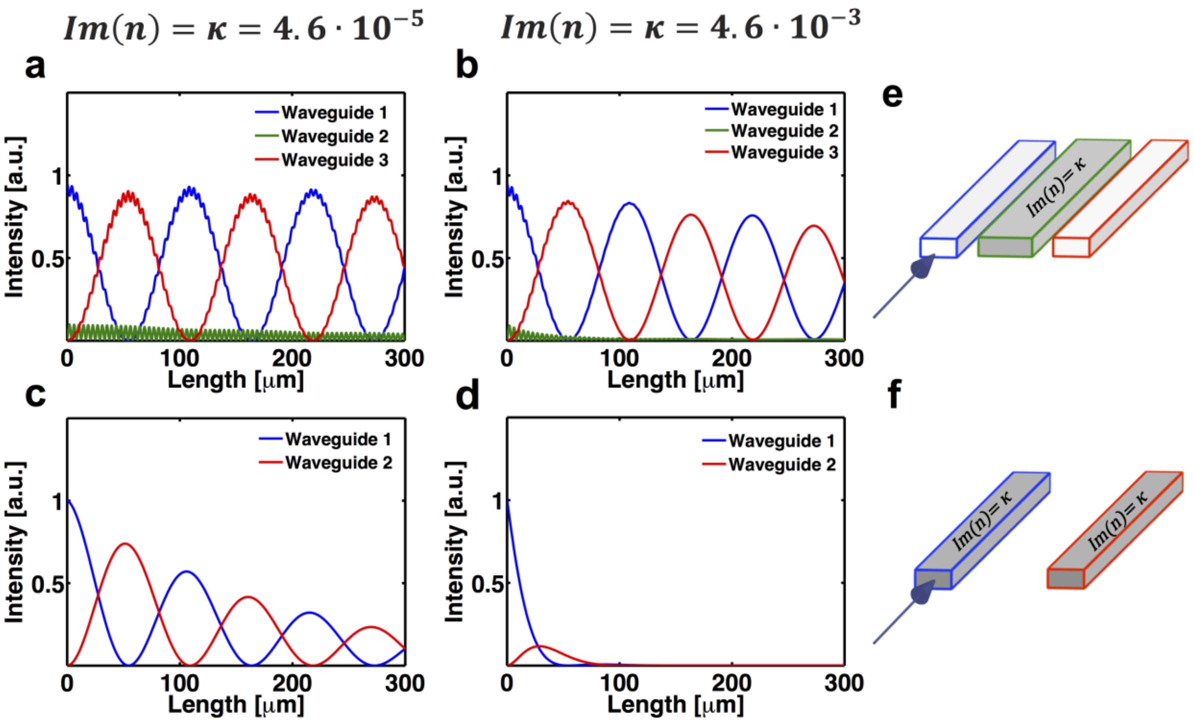

Supplementary Figure 2 - Tolerance to induced-loss in an AE three-waveguides configuration. (a) Simulation of propagation in a three-waveguides AE configuration including loss of $Im(n) = \kappa = 4.6 \cdot 10^{-5}$ in the middle waveguide as evaluated from a generalized NLS model. We observe very minute signal loss since the propagation length of the signal in the middle waveguide is significantly reduced. As a comparison, an equivalent two-waveguide system is calculated under same conditions (c). Here the signal is affected severely from loss due to the direct absorption in the waveguides. (b,d) same configurations as (a,c) but with two orders of magnitude higher loss coefficient $Im(n) = \kappa = 4.6 \cdot 10^{-3}$. The three-waveguides configuration (b) shows a great tolerance to the significantly higher losses experiencing a loss of 0.96 dB whereas no signal is transmitted in the two-waveguides system (d). (e,f) schematic of the simulated three- (e) and two-waveguides (f) configurations.

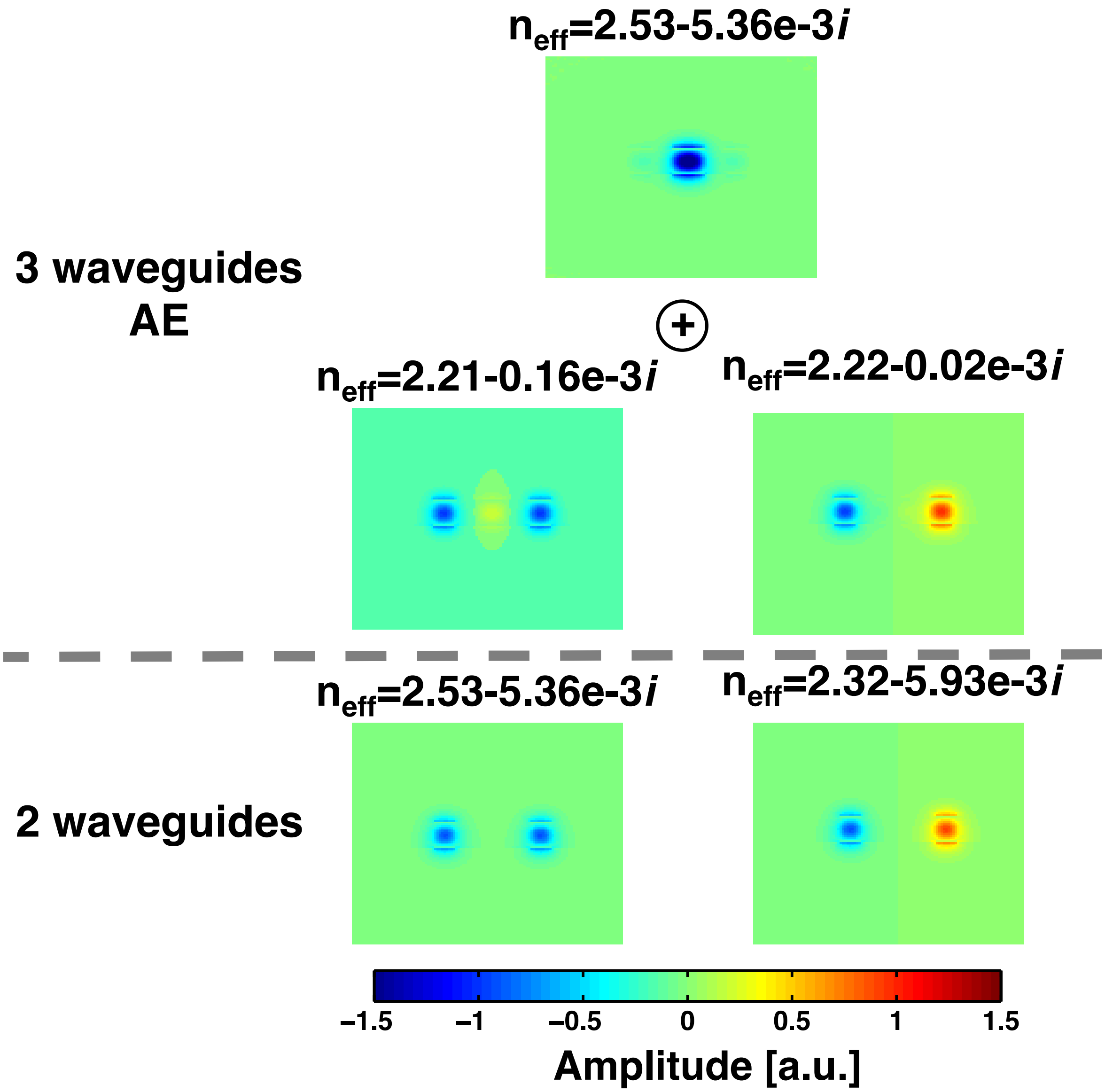

Supplementary Figure 3 - Comparison of the eigenmodes and the effective indices of a three-waveguides AE configuration (top panel) and a two-waveguides configuration (lower panel). The simulation includes loss of $Im(n) = \kappa = 4.6 \cdot 10^{-3}$ in the middle waveguide for the 3 waveguides AE configuration and in both waveguides for the 2 waveguides configuration, as evaluated from a generalized NLS model. (Upper panel) the lower two eigenmodes are the symmetric and antisymmetric modes of the effective two-waveguides coupler achieved by the "2+1" decomposition. We observe that the imaginary part of their effective indices is two orders of magnitude less than the equivalent two-waveguides coupler under same losses (lower panel).

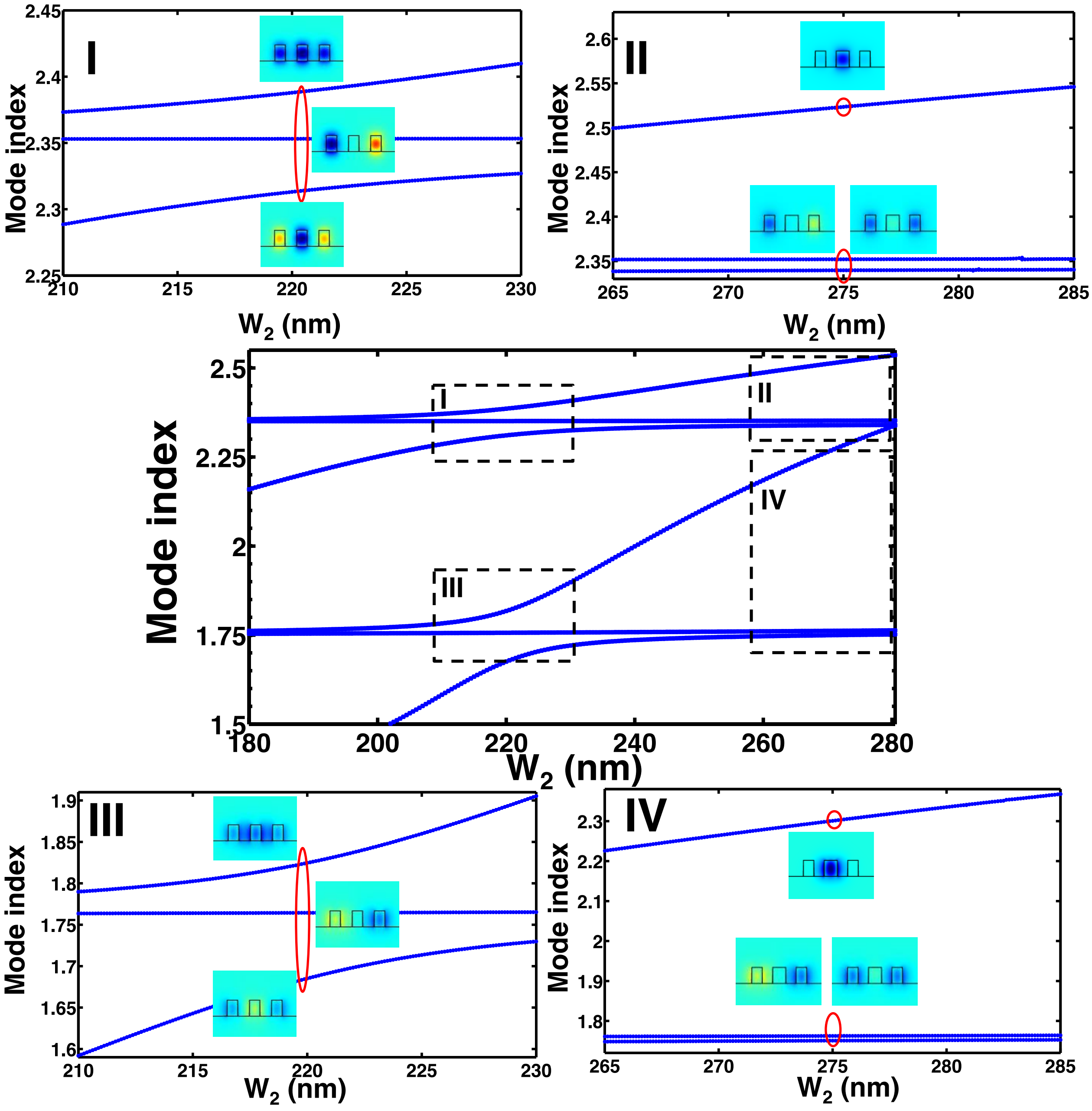

Supplementary Figure 4 – Band diagram of three eigenmodes in 3 coupled waveguide system as a function of varying middle waveguide mode (width) mismatch. I-II upper band TM mode, III-IV lower band TE mode. In both bands the AE decomposition is observed. We see that, as the middle waveguide mismatch increases, the system converges to symmetric and anti-symmetric eigenmodes involving only the outer waveguides whereas the third eigenmode is comprised only of the middle waveguide i.e. it becomes increasingly decoupled.

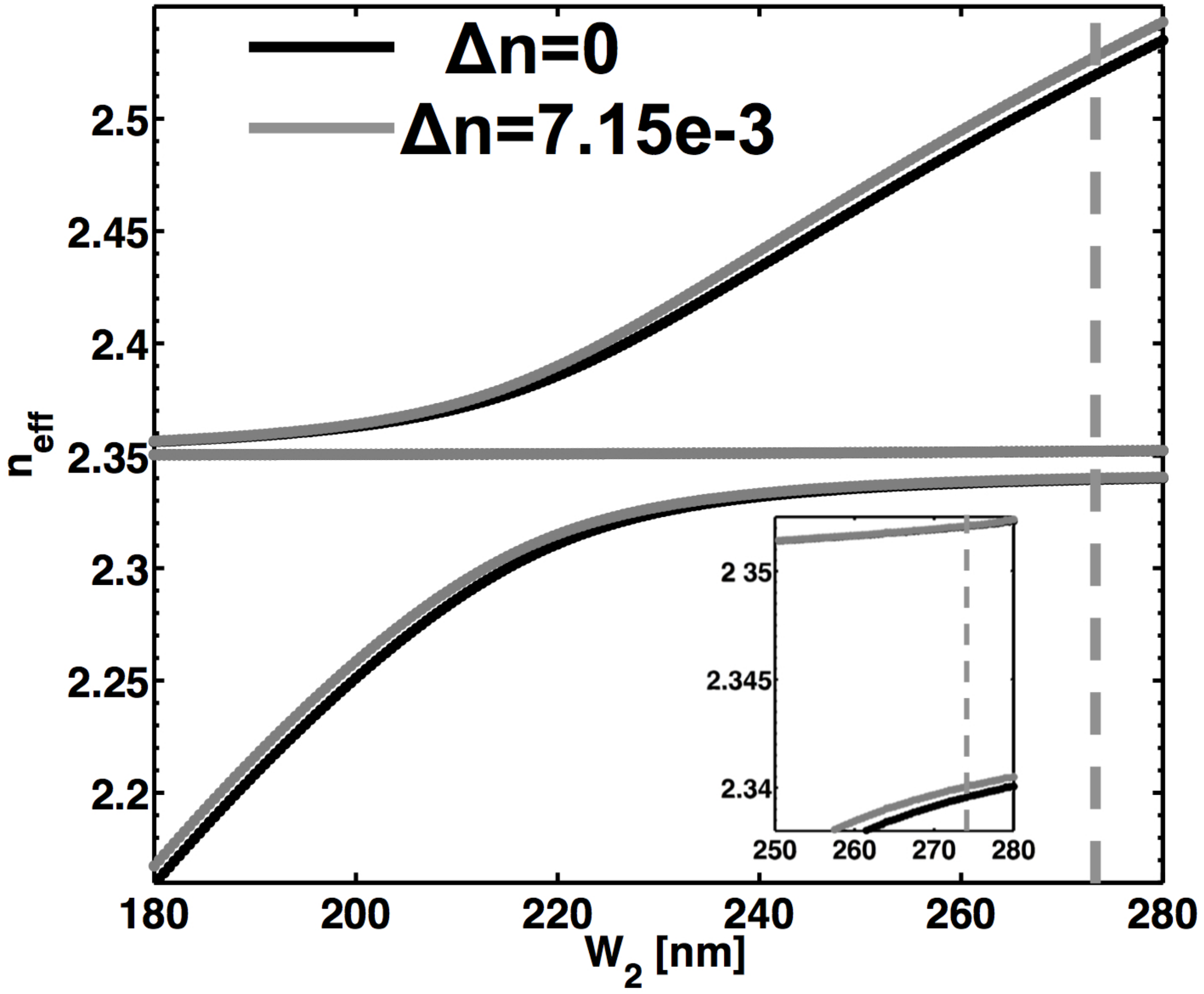

Supplementary Figure 5 – Comparison of band diagrams of the three mode systems. (Solid black curve) With no index change induced in the middle waveguide and (light grey curve) with an index change of $\Delta\boldsymbol{n} = \mathbf{7.15} \times \mathbf{10^{-3}}$. We observe that, although significantly decoupled, the dark mode (upper branch of the diagram) induces an effective index shift in one the effective two-waveguides eigenmodes (inset). This shift ultimately translates into an intensity modulation of the signal transmitted through the AE-induced effective two-waveguides coupler.

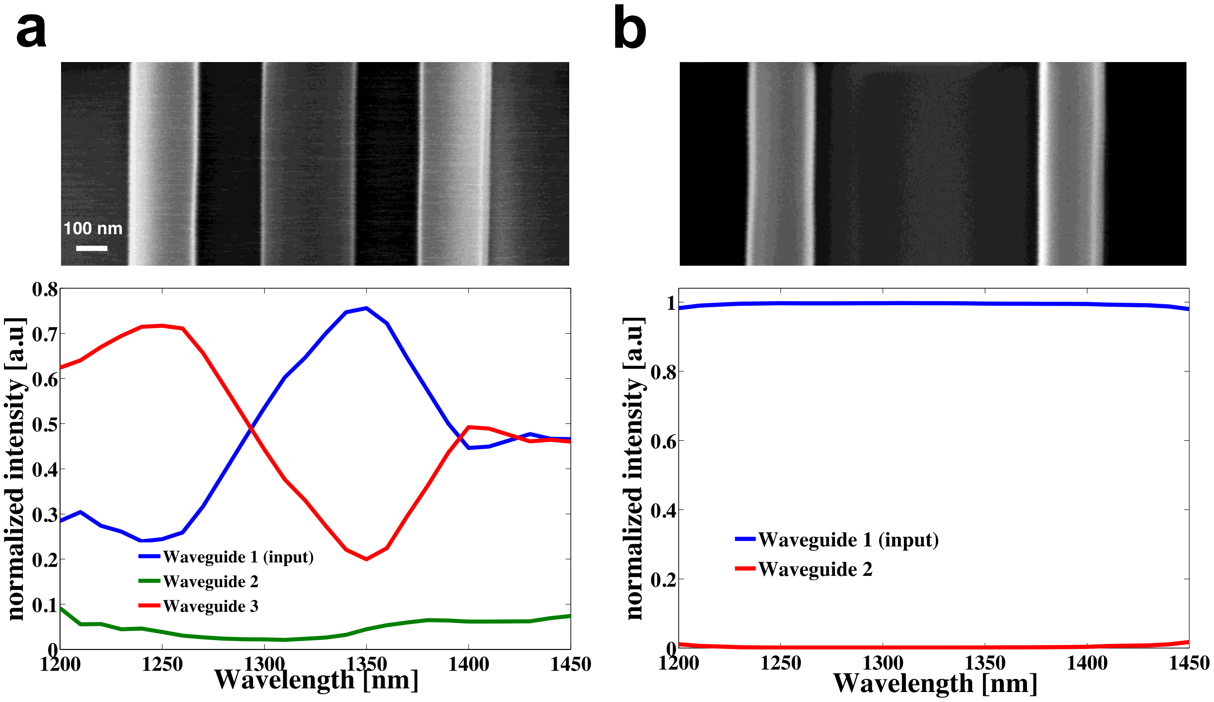

Supplementary Figure 6 – Wavelength dependence of the AE configuration. (a) We clearly observe that the middle waveguide (#2) is eliminated over all the wavelengths swept while the outputs of the outer waveguides (#1 and #3) are varying as function of the wavelength. To further assess that the effect observed is due to the 3 waveguides dynamics we repeat the same experiment on a control sample consisting of two waveguides of same dimensions (220 nm) and with a gap between them identical to the gap (720 nm) between the outer waveguides in AE sample. The results (b) show that in the 2 waveguides case the outputs from the outer waveguides remain fairly constant over the wavelength range scanned. This comparison clearly shows that the dynamics observed on (a) originates from the interplay between V_{13}, V_{12} (V_{23}) and $\Delta\beta$ as expressed in the V_{eff} of the reduced two-waveguide system introduced in the main text.

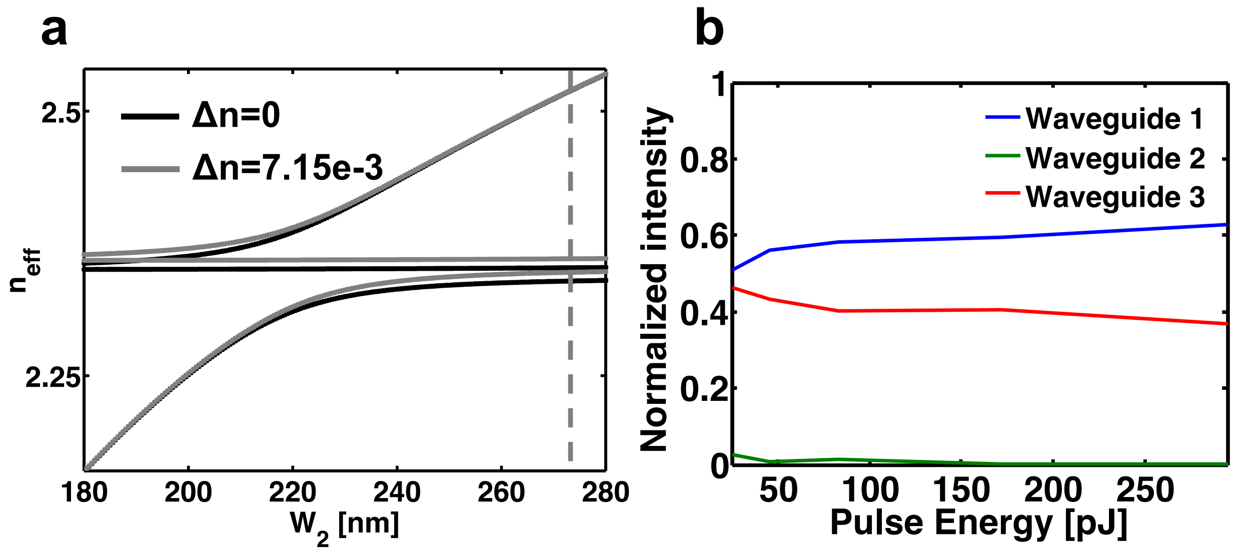

Supplementary Figure 7 - Adiabatic elimination in waveguides as a function of injected signal intensity. (a) Numerical calculation of the band diagram of the three-waveguides AE configuration with $\Delta n = 0$ (black curve) and $\Delta n = 7.15 \times 10^{-3}$ (light grey curve). The signal-induced refractive index change is considered in the two outer waveguides. The 2+1 decomposition characteristic of the AE in waveguides is clearly preserved even under signal-induced refractive index change. Our tested AE configuration is indicated by the grey horizontal dashed line. (b) Experimental verification of the AE behavior versus signal energy. The plot represents the relative output intensity of the three waveguides under increasing input signal energy. The signal is injected into waveguide 1. We clearly observe that the overall AE 2+1 decomposition is preserved over a broad range of input energy where most strikingly the waveguide 2 remains “dark”. The relative intensity change between waveguide 1 and 2 arises from the relative phase shift due to signal-induced refractive index change.

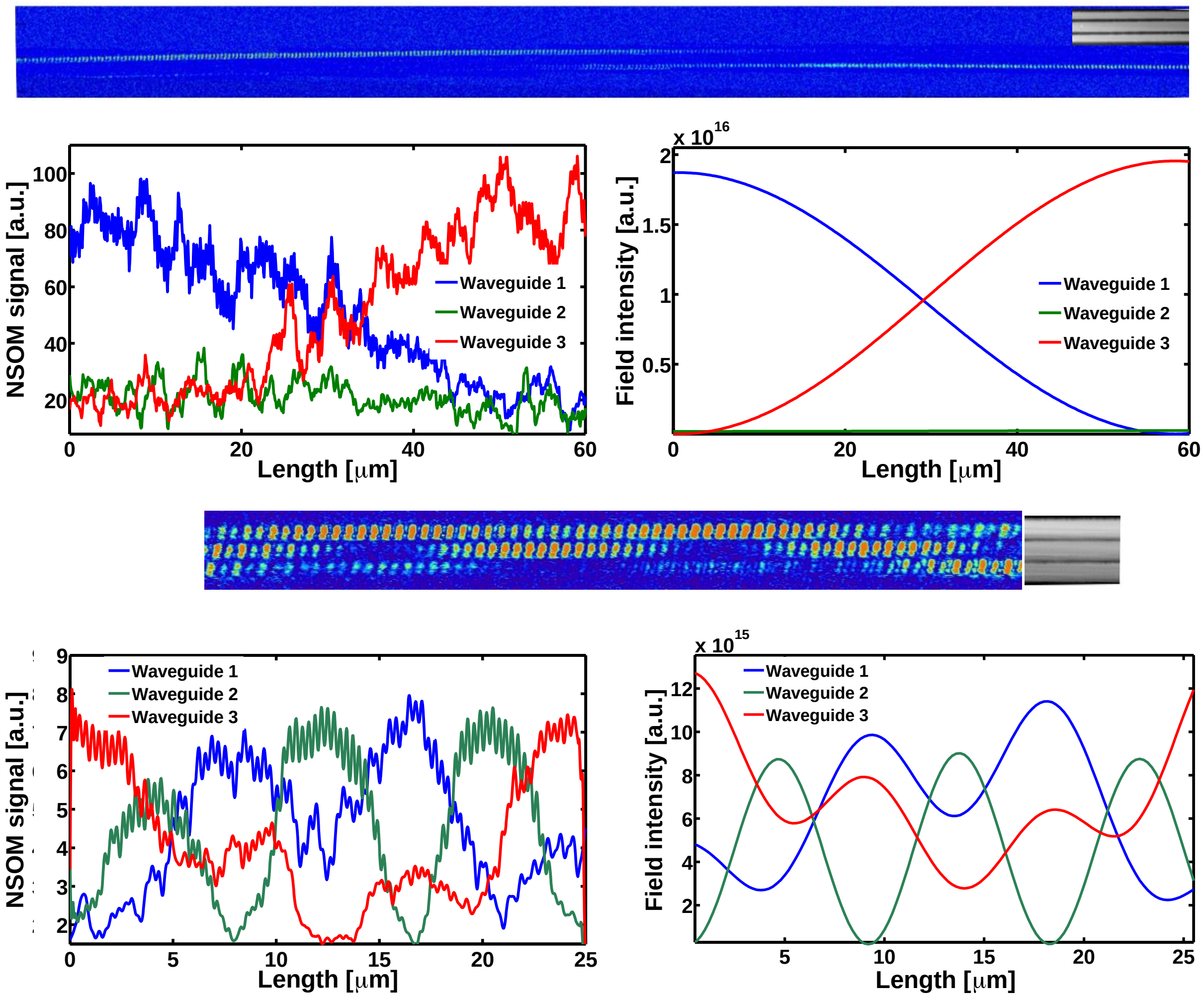

Supplementary Figure 8– NSOM measurements of adiabatic elimination in waveguides. We clearly observe that in the AE configuration (a-b) no detectable signal from the middle waveguide along the 100 μm propagation was recorded confirming that the latter is indeed "eliminated". The control case, (d-e), shows clearly that all the waveguides are populated. In such configurations, we have verified that the Stark shift is negligible, thus $\boldsymbol{\Delta\beta_{eff} = 0}$ (as $\boldsymbol{\Delta\beta_{12} = -\Delta\beta_{23}}$ and $\boldsymbol{\Delta\beta_S \rightarrow 0}$). In all cases, there is an excellent agreement between the NSOM measurements and the numerical simulations (c,f). The dimensions used in the numerical simulations were taken from SEM measurements of the AE and control sample.

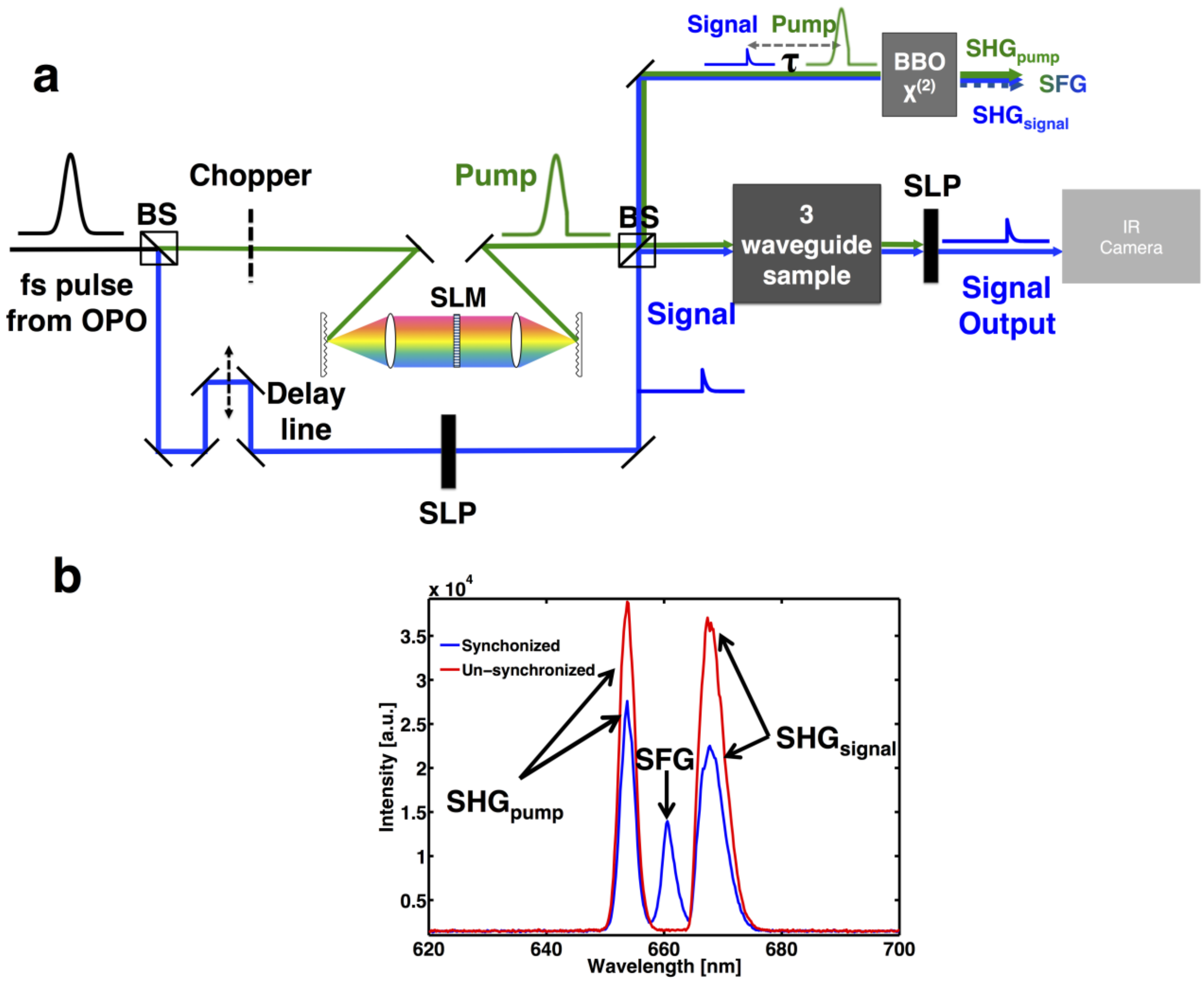

Supplementary Figure 9 – Pump and signal synchronization for AE coupling control (a) Experimental setup scheme showing the auxiliary beam path for synchronization of ultrafast pump and signal beams (b) Nonlinear generations in the BBO crystal due to the presence of pump and signal beams. Each beam generates its own second harmonic generation (SHG) through the $\chi^{(2)}$ of the BBO a crystal('un-synchronized' spectrum). However when the pump and signal pulses are present at the same time in the crystal, they also mix through the $\chi^{(2)}$ nonlinearity to give a sum-frequency generation (SFG) ('synchronized' curve). We use the SFG signal as the indication of synchronization.

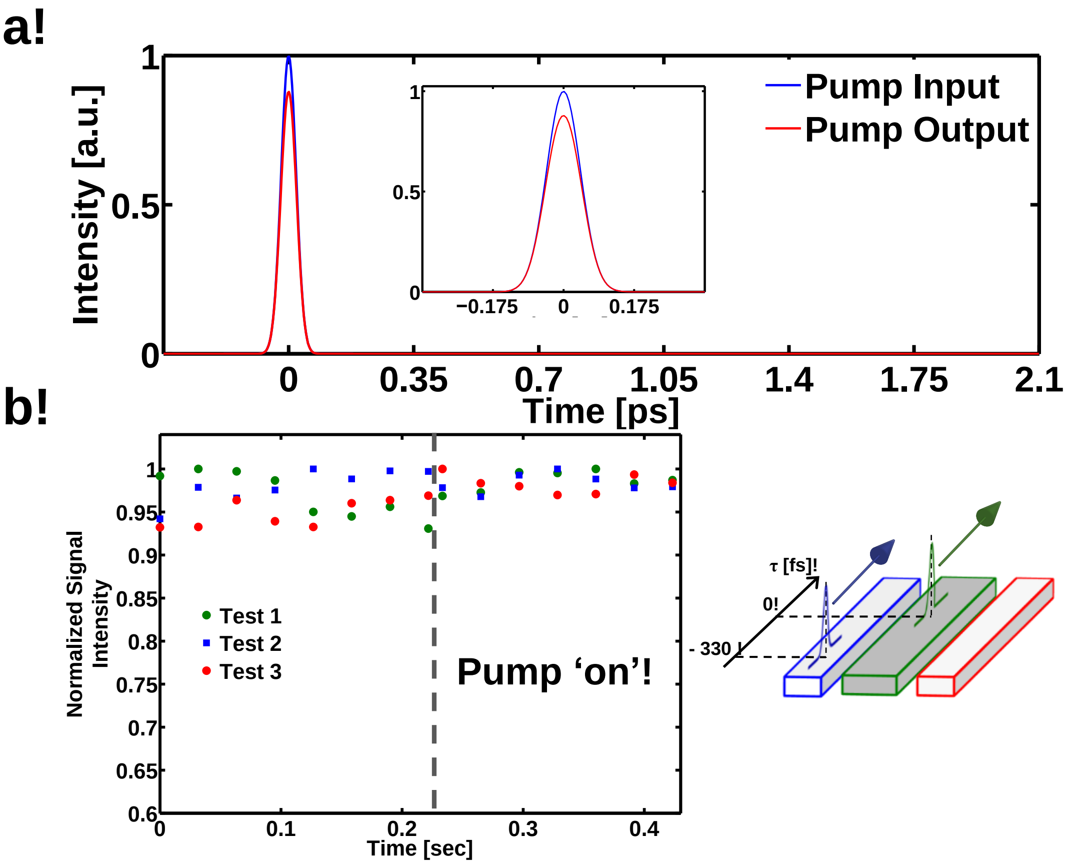

Supplementary Figure 10: Free carriers effects on the AE modulation scheme – (a) results from the modified NLSE showing no temporal distortion due to FC after propagation of the 400 W peak power pump pulse in the middle waveguide (blue curve- Pump beam at the input; red curve- Pump beam at the output). (b) Signal output from waveguide 1 when pump beam is inserted in middle waveguide 330 fs before the signal enters waveguide 1. No modulation is observed which confirms experimentally that no long-lived Free Carriers (FC) effect is involved in the index modulation mechanism.

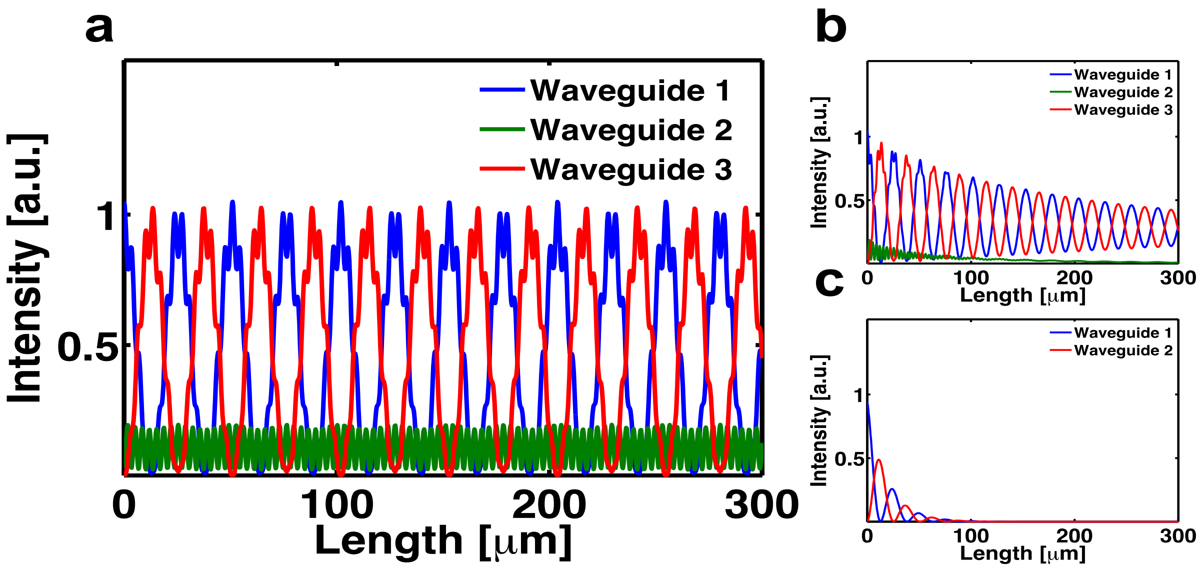

Supplementary Figure 11 - Adiabatic elimination with middle waveguide near cutoff. (a) The signal oscillates fast between the outer waveguides because of the increased effective coupling. The fast oscillations make the configuration more sensitive to modulation of the middle waveguide. (b) Propagation of the signal in the same configuration taking into account losses in the middle waveguide. However comparison with a similar standard two-waveguides configuration under the same loss (c) shows that the AE configuration still offers superior tolerance to losses.

Supplementary Table

Supplementary Table 1: Performance comparison between AE modulator and established modulation techniques

Structure	Mach-Zehnder interferometer	Mach-Zehnder interferometer	Ring	Disk	Ring	Adiabatic elimination
**Modulation principle**	Depletion of a horizontal p-n junction [Supp. Ref.5]	Forward biased diode [Supp. Ref. 6]	Forward biased diode [Supp. Ref. 7-8]	Reverse-biased p-n junction [Supp. Ref. 9]	Reverse-biased p-n junction [Supp. Ref. 10]	Potentially depletion of a p-n junction
**Device footprint [μm^2]**	~1x10^4	~1x10^3	~1x10^2	20	~1x10^3	~2x10^2
**Energy per bit [fJ/bit]**	~3x10^4	~5x10^3	~300	85	50	~8x10^3
**DC modulation depth/insertion loss (dB)**	>20/~7	6-10/~12	>10/<0.5	8/1.5	6.5/2	20/~2 (potentially around 0.2)
**Working spectrum**	>20	-	~0.1	~0.1	0.1	broadband
**Thermal stability**	Good	Good	Very poor	Very poor	Very poor	Good

Supplementary Discussion

Supplementary Discussion 1: Rigorous analysis of adiabatic elimination in integrated photonics

The general evolution of the electromagnetic field in a linear three mode directional couplers is determined by the following three coupled mode equations [31-32]:

$$\frac{d}{dz}\begin{pmatrix} A_1 \\ A_2 \\ A_3 \end{pmatrix} = i\begin{pmatrix} 0 & V_{12}e^{-i\Delta\beta_{12}z} & V_{13}e^{-i\Delta\beta_{13}z} \\ V_{12}^{*}e^{i\Delta\beta_{12}z} & 0 & V_{23}e^{-i\Delta\beta_{23}z} \\ V_{13}^{*}e^{i\Delta\beta_{13}z} & V_{23}^{*}e^{i\Delta\beta_{23}z} & 0 \end{pmatrix}\begin{pmatrix} A_1 \\ A_2 \\ A_3 \end{pmatrix} \qquad (1)$$

The mathematical procedure behind adiabatic elimination scheme is the assumption that $\frac{dA_2}{dz} \rightarrow 0$, meaning that the effective field accumulation along the middle waveguide will be negligible along the propagation. Using such assumption, on the general dynamical equations of the process, seen in Eqs. 1, $i\frac{dA_2}{dz} = V_{12}^{*}e^{-i\Delta\beta_{12}z}A_1 + V_{23}e^{i\Delta\beta_{23}z}A_3$, and integrating it, assuming constant coupling and phase-mismatch, we find:

$$A_2(z) = -i\int dz\,(V_{12}^{*}e^{-i\Delta\beta_{12}z}A_1 + V_{23}e^{i\Delta\beta_{23}z}A_3) = -i\left(\frac{V_{12}^{*}}{-i\Delta\beta_{12}}e^{-i\Delta\beta_{12}z}A_1 + \frac{V_{23}}{i\Delta\beta_{23}}e^{i\Delta\beta_{23}z}A_3\right)$$

$$A_2(z) = \frac{V_{12}^{*}}{\Delta\beta_{12}}e^{-i\Delta\beta_{12}z}A_1 - \frac{V_{23}}{\Delta\beta_{23}}e^{i\Delta\beta_{23}z}A_3$$

In order to keep low excitation value of the middle waveguide, one should assume that the two phases oscillate fast $|\Delta\beta_{12}z| \ll 1, |\Delta\beta_{23}z| \gg 1$, and that the amplitude are relatively small $\frac{V_{12}^{*}}{\Delta\beta_{12}} \ll 1, \frac{V_{23}}{\Delta\beta_{23}} \ll 1$. Under these conditions, the oscillating terms in Eq. (1) vary much faster than the fields' amplitudes at ω_1 and ω_3, allowing us to write an approximation for the intermediate frequency complex amplitude:

$$\frac{dA_1}{dz} = \frac{V_{12}^{*}V_{12}}{\Delta\beta_{12}}A_1 + \left(V_{13} - \frac{V_{12}V_{23}}{\Delta\beta_{23}}\right)e^{i\Delta\beta_{13}z}A_3$$

$$i\frac{dA_3}{dz} = \left(V_{13}^{*} + \frac{V_{23}^{*}V_{12}^{*}}{\Delta\beta_{12}}\right)e^{-i\Delta\beta_{13}z}A_1 - \frac{V_{23}^{*}V_{23}}{\Delta\beta_{23}}A_3$$

Using the following expression $E_1 = A_1(z)exp\left[-i\left(\frac{\Delta\beta_{eff}}{2} - \frac{V_{eff}}{\Delta\beta_{12}}\right)\right]$, an$E_3 = A_3(z)exp\left[-i\left(\frac{\Delta\beta_{eff}}{2} - \frac{V_{eff}}{\Delta\beta_{12}}\right)\right]$, the evolution is written in the rotating frame:

$$\frac{d}{dz}\begin{pmatrix} E_1 \\ E_3 \end{pmatrix} = i\begin{pmatrix} \frac{\Delta\beta_{eff}}{2} & V_{13} - \frac{V_{12}V_{23}}{\Delta\beta_{23}} \\ V_{13} + \frac{V_{12}V_{23}}{\Delta\beta_{12}} & -\frac{\Delta\beta_{eff}}{2} \end{pmatrix}\begin{pmatrix} E_1 \\ E_3 \end{pmatrix}$$

$$\Delta\beta_{eff} = \Delta\beta_{12} + \Delta\beta_{23} + \frac{V_{12}V_{21}}{\Delta\beta_{12}} + \frac{V_{23}V_{32}}{\Delta\beta_{23}}$$

$$V_{eff} = \sqrt{\left(V_{13}^* + \frac{V_{23}^*V_{12}^*}{\Delta\beta_{12}}\right)\left(V_{13} - \frac{V_{12}V_{23}}{\Delta\beta_{23}}\right)}$$

$$E_m = \frac{|V_{12}|^2}{2\Delta\beta_{12}} - \frac{|V_{23}|^2}{2\Delta\beta_{23}}$$

In contrast to the analysis, which has been performed in other research fields, in the integrated photonics case a direct V_{13} coupling coefficient between the outer waveguides may become significant. This coupling acts simultaneously with the conventional nearest neighbor coupling between the waveguides, leading to an effective reduced SU(2) dynamics between the two outer waveguide system in the general SU(3) dynamical system.

The effective two-level system can be designed by changing the geometry and the dimensions of the waveguides. Examples of such manipulation can be viewed in Supplementary Figure 1, showing the V_{13} dynamical evolution for different relations between the direct coupling parameter V_{13} and the indirect coupling $\frac{V_{12}V_{23}}{\Delta\beta_{23}}$.

Supplementary Discussion 2: Effect of losses in the AE scheme

The AE scheme holds also in case the middle waveguide is lossy, either by a linear loss, which originated from the imaginary part of the refractive index (as in the case of plasmonic and near resonant interactions), or by a nonlinear loss, which originate from the imaginary part of a nonlinear susceptibility (as in the case of TPA in Silicon Photonics). In such case, the mode index of the middle waveguide will be modified to include an imaginary part, as follow: $\beta_1 = \frac{2\pi}{\lambda} n_1^{\text{eff}},\ \beta_2 = \frac{2\pi}{\lambda} n_2^{\text{eff}} + \mathrm{i}\gamma_2, \beta_3 = \frac{2\pi}{\lambda} n_3^{\text{eff}}$. In this case both of the mode index and the coupling coefficient will become complex valued. Nonetheless, when employing the adiabatic elimination procedure, due to the fact that the procedure cause minimization of light propagation via the middle waveguide (the rate of light transfer to the middle waveguide is equal to the rate of transfer outside the middle waveguide), the effective loss coefficient in the reduced two-level dynamics will be reduced by several order of magnitudes (which will be proportional to the real value of phase-mismatch, i.e to $\left(n_2^{\text{eff}} - n_1^{\text{eff}}\right)\omega/c$).

In order to confirm this behavior, we have considered and simulated a couple of cases: One with an imaginary part of the refractive index of $Im(n) = \kappa = 4.6 \cdot 10^{-5}$ estimated from a time-dependent generalized Non-linear Schrödinger (NLS) model [Supplement Figure 10 , Supplement discussion 7] with the exact experimental parameters that we experienced in our experiments, and the other with an extreme case where the losses are 2 orders of magnitude higher than the loss in our experiment i.e. $Im(n) = \kappa = 4.6 \cdot 10^{-3}$. The losses in our experiment were induced by the strong pump through Two Photon Absorption (TPA) and Free Carrier Absorption (FCA). However the analysis presented here is valid for other loss mechanisms such as originated by metallic contacts or very lossy middle plasmonic waveguide.

In the numerical analysis, we have simulated the propagation of light injected in waveguide #1 (outer waveguide), and the loss (induced TPA and FCA) was considered for the pump only. For comparison, we considered also a two-waveguides case where the loss is considered in both waveguides since the intense pump oscillates between the 2 waveguides and induce TPA and FCA in both waveguides. For the three-waveguide AE case, the loss is considered in the middle waveguide, as the intense pump propagates only along the middle waveguide.

As can be clearly seen from Supplementary Figure 2(a-b), the effective loss experienced by the signal in the AE configuration is significantly reduced as compared to the standard two-waveguides coupler seen in Supplementary Figure 2(c-d). ***This is despite the fact that the same loss coefficient is considered in both cases.*** The physical origin for the lower loss is related to

the nature of the AE evolution, in which *the rate of light transfer to the middle waveguide is equal to the rate of transfer from the middle waveguide*, meaning that their effective propagation there is significantly reduced as the photons do not propagate much in the middle waveguide and are not being effected by the loss in the middle waveguide. For this reason the adiabatic elimination of the lossy middle waveguide dramatically reduces the overall loss of the system while still allowing coupling control through the middle waveguide.

To complete the picture, we compare the eigenmodes of the two systems, as presented in Supplementary Figure 3. We clearly observe that both eigenmodes of the lossy two-waveguides coupler exhibit comparable imaginary part in their effective mode indices, since the intense pump pulse that induces the nonlinearity propagates in both waveguides, whereas in the three-waveguides AE configuration, the imaginary part of the **effective** mode indices of the reduced two-waveguides system are smaller by 2 orders of magnitude.

These simulations clearly indicate that the energy is oscillating between waveguides 1 and 3, despite tunneling through the lossy middle waveguide, doesn't experience significant loss as compared to an equivalent lossy 2-waveguide coupler.

Supplementary Discussion 3: Band diagram of three mode dynamics in SOI waveguides

Visualizing adiabatic elimination regime in three coupled waveguides system can be seen by calculating the band diagram of the eigenmodes of the system. Here we extend the band diagram given in Fig. 3 for the TM mode, to include also the TE modes of the configurations. As seen, in Supplementary Figure 4, the TE mode has the same characteristics as the TM modes and the adiabatic elimination procedure holds both for TM and TE modes.

We show in Supplementary Figure 5 how the band diagram method can be used to visualize also the modulation process. We see that under modification of the refractive index of the middle waveguide, there is change of effective index induced to one of the effective two waveguides eigenmodes in the decomposed configuration (see inset in Supplementary Figure 5). This induced index change will translate in the modulation of the output signal intensity as we have shown in the main text.

Supplementary Discussion 4: Broadband response of AE scheme

To demonstrate the broadband character of the AE scheme in waveguides we performed a wavelength scanning experiment on the sample shown in Fig 2a. We have used a Ti:Sa pumped OPO (Coherent/APE Chameleon Compact OPO) as our pump source using the far field setup described in the main text Methods, with a sub μW average power to avoid any nonlinear effect in the waveguides. The wavelength of the OPO was tuned from 1200 nm to 1450 nm and the outputs from the different waveguides was acquired in the same fashion as described in the Methods. The results of this wavelength sweep are depicted in Supplementary Figure 6(a). We clearly observe that the middle waveguide (#2) is eliminated over all the wavelengths swept while the outputs of the outer waveguides (#1 and #3) are varying as function of the wavelength. To further assess that the effect observed is due to the 3 waveguides dynamics we repeat the same experiment on a control sample consisting of two waveguides of same dimensions (220 nm) and with a gap between them identical to the gap (720 nm) between the outer waveguides in AE sample. The results (Supplementary Figure 6(b)) show that in the 2 waveguides case the outputs from the outer waveguides remain fairly constant over the wavelength range scanned. This comparison clearly shows that the dynamics observed on Supplementary Figure 6(a)

originates from the interplay between V_{13}, V_{12} (V_{23}) and $\Delta\beta$ as expressed in the V_{eff} of the reduced two-waveguide system introduced in the main text.

Supplementary Discussion 5: Effect of third order nonlinearity on the signal field.

We discuss here the effect of the strong third order nonlinearity on the signal field. The discussion contains further experimental results and numerical calculations. As we show below, the presented AE 2+1 decomposition still holds with a strong signal field that excite third order nonlinearity and change the index of the outer waveguides.

The AE dynamics, by its nature, decomposes three coupled waveguides system into two coupled outer waveguides (two separate symmetric and antisymmetric eigenmodes) and an uncoupled middle waveguide (an eigenmode that consists only of the middle-waveguide). These modes have very distinct mode-indices, thus, such 2+1 decomposition is very robust to small modifications of the mode index due to third order nonlinearity of the signal, which is at most a few percent. In order to confirm this model, we have performed both COMSOL numerical simulations and experimental verification which results are presented in Supplementary Figure 7. Our simulations show that nonlinearity in the outer waveguides indeed causes a shift of index of the outer waveguides. However such index shift causes only a minor change of the mode-indices of the coupled system and doesn't break the AE decomposition Supplementary Figure 7(a).

This is further confirmed in experimental measurements that were conducted on our three-waveguides AE configuration, where we have measured the relative intensity in each waveguide as a function of the signal's pulse energy, which was always coupled entirely to the outer waveguide #1. In this experiment, the output of our OPO was directly coupled to the waveguide #1 without any splitting into two beams and going through the pulse shaper (as done in the modulation experiment presented in Fig. 4). This allowed us to increase the energy coupled into the waveguide to the maximum available pump energy. We observe that, while the ratio between the outer waveguides varies slightly as a consequence of nonlinear modulation in the real part of the refraction index, the overall 2+1 decomposition is preserved over a wide range of pulse energies. The experimental data is presented in Supplementary Figure 7(b). This further strengthens the robustness of the AE procedure in waveguides we already presented in Supplementary Figure 6 as a function of wavelength.

Moreover we considered also how the nonlinear induced losses affect the signal propagation in the outer waveguides. This aspect is related to the high TPA and subsequent FCA that an intense

signal will induce as it propagates. The strong TPA will cause a decrease in the signal intensity exactly as in a two-waveguides system (shown in Supplementary Figure 2). The signal will nonetheless remain in the outer waveguides due to the robustness of the AE mechanism.

Supplementary Discussion 6: NSOM scan of Adiabatic Elimination and propagation simulation

We acquired NSOM images along the waveguides in both AE configuration (Supplementary Figure 8 (a-b)) and the control configuration (Supplementary Figure 8(d-e)) and compared with numerical simulations. The results in Supplementary Figures 8 (a-b) show clearly that in the AE configuration no detectable signal from the middle waveguide along the 100 μm propagation was recorded confirming that the latter is indeed "eliminated". In the control case, the results Supplementary Figures 8(d-e) show clearly that all the waveguides are populated. In such configurations, we have verified that the Stark shift is negligible, thus $\Delta\beta_{eff} = 0$ (as $\Delta\beta_{12} = -\Delta\beta_{23}$ and $\Delta\beta_S \rightarrow 0$). In all cases, there is an excellent agreement between the NSOM measurements and the numerical simulations Supplementary Figures 8(c,f). The dimensions used in the numerical simulations were taken from SEM measurements of the AE and control sample. The simulations are based on an eigenmodes decomposition and propagation. The eigenmodes of the waveguide system are obtained using COMSOL multiphysics.

Supplementary Discussion 7: Time dependent evolution of the pump beam and free carriers effects on the AE modulation scheme

We have investigated the influence of the TPA and the generated free carriers from both a simulation and experimental point of view. For the simulation aspect we have implemented the modified Non-Linear Schrödinger equation [Supplementary reference 1,2] to include the TPA and FCA induced by the pump beam as it propagates in the middle waveguide.

$$\frac{\partial A}{\partial z} + \frac{\alpha_l}{2} A = i\kappa |A|^2 A - \frac{\alpha_f}{2} A \qquad (2)$$

In our simulations, and in accordance with previous works, we have neglected the dispersion effects. This is because the group velocity dispersion GVD in silicon wires although significantly enhanced as compared to bulk Silicon will not induce a sensible temporal distortion over short distance [Supplementary reference 3]. In our case we estimate that the 140 fs pulse over a 300 μm propagation length will broaden only to no more than 150 fs. A is the slowly varying amplitude of the pulse, α_l is the linear loss and $\kappa = \gamma + \frac{i\Gamma}{2}$. Γ is related to the TPA coefficient $\Gamma = \frac{\beta_{TPA}}{A_{eff}}$ with $\beta_{TPA} = 5 \times 10^{-12}\, m/W$ [Supplementary reference 1,2] and A_{eff} is, in our case, calculated to be using the definition in [Supplementary reference 2] $A_{eff} = 0.11\, \mu m^2$. FCA is included by $\alpha_f = \sigma N$, where $\sigma = 1.45 \times 10^{-21}\, m^2$ for silicon [Supplementary reference 2] and N is the density of carriers produced by TPA. It is obtained by solving [Supplementary reference 2]

$$\frac{\partial N}{\partial t} = \frac{\beta_{TPA}}{2h\upsilon_0} \frac{|A|^4}{a_{eff}^2} - \frac{N}{\tau_c} \qquad (3)$$

Where τ_c is the effective carrier lifetime and taken to be $\tau_c = 0.5\, ns$. We thus consider that the repetition rate is low enough (80 MHz) that free carriers produced by one pulse have enough time to recombine before the next pulse arrives. This simplification allows us to focus on a single pulse. The injected peak power is taken to be 400 W. As we can see from the Supplementary Figure 10(a), the output pulse follows the input pulse shape. This means that the FCA effect is negligible since under non-negligible FCA: "free carriers created by the leading edge of a pulse would absorb light in the pulse tail. As a result, FCA will dampen the pulse tail but leave intact the leading part of a pulse" [Supplementary reference 2]. This would result in pulse distortion as observed in fig. 6 in Supplementary reference 1. As shown in Supplementary reference 1, it follows therefore that under our experimental conditions, the fast Kerr nonlinearity dominates

the slow FC effect. This is further confirmed experimentally by inserting a delay between the pump and the signal. An index modulation originating from FC would be long-lived and exponentially decaying following the recombination decay of the pump-generated FC. This exponentially decaying index modulation would influence the output signal during a timescale of about τ_c. This scenario is unequivocally ruled out by the result presented in Supplementary Figure 10(b). We delay the signal behind the pump by only 330 fs and we observe no modulation of the signal. This clearly shows that the underlying modulation mechanism is ultrafast and can't be attributed to FC.

Supplementary Discussion 8: More efficient AE modulation and comparison to other modulation scheme in integrated photonics

In our current demonstration of modulation shown in Fig. 4, we had two limitations: (1) we have used a fs-oscillator pulse with finite power that was split into two synchronized pulses and (2) our will to avoid the inherent high TPA nonlinearity in Silicon (which occur in high intensity and can influence the speed of the device with the subsequent FCA). By using higher laser fluence, we could achieve higher contrast, and even full 1–to-0 transition. This is due to the fact that the change of $\Delta\beta$ (our control) scales approx. linearly with the increase of intensity:

$$V_{\text{eff}} = \frac{2\pi}{L_{\text{inv}}} = V_{13} - \frac{V_{12}^2}{\Delta\beta} \rightarrow L_{\text{inv}} = \frac{2\pi}{V_{13} - \frac{V_{12}^2}{\Delta\beta}} \sim \frac{2\pi\Delta\beta}{V_{12}^2}; \;\; L_{\text{inv}}^{\text{NL}} \sim \frac{2\pi(\Delta\beta_0 + \Delta\beta^{\text{NL}})}{V_{12}^2} \;;$$

$$\Delta\beta = \frac{2\pi(\Delta n + n_2^{\text{kerr}} I)}{\lambda} = \Delta\beta_0 + \Delta\beta^{\text{NL}}$$

The several degrees of freedom available in the AE scheme to tune the effective coupling between the outer waveguides can be further exploited to improve the extinction ratio and to reduce the power needed. A particularly promising direction is to design the device such that the middle waveguide is near cutoff. By doing so, the device operates in the regime presented in Supplementary Figure 1(d) where the signal rapidly oscillates between the outer waveguides and then becomes much more sensitive to index change in the middle control waveguide. To exemplify this approach, we have simulated such device where the outer waveguides have the same dimensions as in the device presented here ($W_1 = W_3 = 220\ nm$).

Whereas the middle waveguide is near cutoff ($W_2 = 170\,nm$. In order to meet the right condition ($V_{13} > \frac{V_{12}^2}{\Delta\beta}$), we reduce the overall gap to 470 nm to increase the V_{13} coupling. The signal injected in waveguide 1 oscillates quickly between 1 and 3 as shown in the Supplementary Figure 11. The fast oscillations make indeed the device more sensitive to index change in the middle waveguide and our simulation shows that an extinction ratio of 20 dB is achieved in such configuration with a change of index of $\Delta n = 9 \times 10^{-4}$ requiring only 8 pJ, ***9 times less*** than in the current configuration.

Moreover, the advantage of the AE scheme in this regime over standard two-waveguides system in the presence of losses is still evident from the comparison presented in Supplementary Figure 11 (b-c). A further improved performance, such as higher extinction ratio with sub pJ consumption can be achieved with a dedicated optimization of the degrees of freedom (couplings, phase mismatch and polarization).

For a performance comparison we refer here to Supplementary Reference 4, which provides a comparison between several metrics related to modulators. We present in Supplementary Table 1 an adaptation to include the AE device. We consider the simulated device above, which offers improved performances, even if, as we stressed, there is still much room for optimization.

As noted in Supplementary Reference 4:

"*One can see a trade-off emerging from the data [...] between power consumption, optical spectrum, and device footprint. Resonators have power consumption close to the expected target required on the order of 10 fJ per bit, as well as a very small footprint. However, they suffer from a narrow-spectrum bandwidth and tight fabrication tolerances. Furthermore, the thermal tuning power required to stabilize them at a predefined frequency of operation must be included in estimating the total system power requirement. Interferometers, however, have much more relaxed fabrication tolerances, wider operating spectra, and are less dependent on temperature variations. Nevertheless, they suffer from a larger footprint that increases their power consumption*."

As we can see, the AE modulation scheme has much to offer in the trade-off outlined here. The scheme we propose here outperforms the MZI in most of the parameters by offering a much reduced insertion loss and a smaller footprint. At the same time, while offering a footprint comparable to rings resonator, the AE modulator is broadband and doesn't suffer from the thermal stabilization issues that plague resonance-based modulators.

Supplementary Methods

Supplementary Methods 1: Pump and signal synchronization for AE coupling control

We describe here the method used to synchronize the pump and the signal injected in the AE modulation experiment described in the text and in Fig. 3. In the setup we focus (10x Objective) the shaped pump and the truncated signal beams onto a 2 mm thick BBO crystal (Caston Inc.) cut to be phase matched for Second Harmonic Generation (SHG) at 1310 nm. The pump and signal beams are collinear and spatially overlap on the crystal input facet. The output from the crystal is collected to a spectrometer (Ocean Optics USB4000) as shown in Supplementary Figure 9(a). As expected from the $\chi^{(2)}$ nonlinearity of the BBO we observe the SHG from both the truncated pump and the truncated signal. The SHG generated are spectrally offset owing to the slight spectral difference between the beams. When the pump and signal pulses enters the crystal at the exact same time, in addition to the regular SHG peaks we expect a Sum Frequency Generation (SFG) which is observed in between the two SHGs, see Supplementary Figure 9 (b). The appearance of the SFG is thus a signature of the synchronization. We thus scan the delay line to find an optimized SFG signal.

Supplementary Methods 2: Step by step experimental procedure for the active control exp. (Fig. 4)

In the following, we give a detailed explanation on the single-shot pump-probe apparatus used to obtain the results presented in Fig. 4 in the main text. We will start by detailing the apparatus of the single-shot measurements (which is used to measure nonlinear spectral information in many research areas and for various samples, such as coherent Raman processes in atomic, molecular and biological systems).

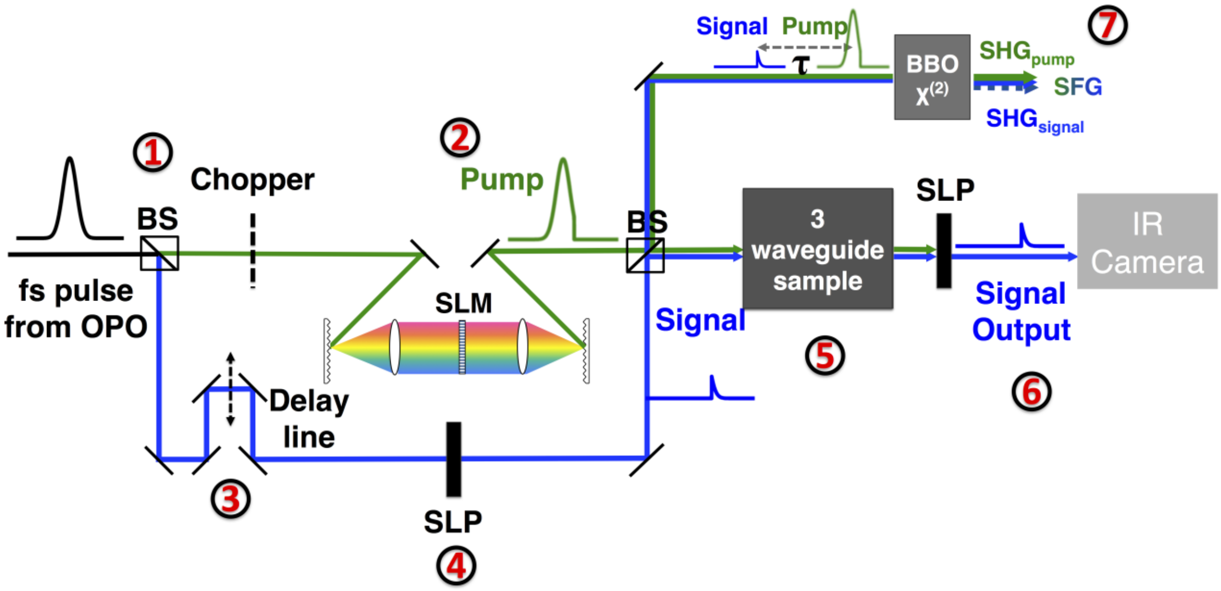

The setup is reproduced in the figure above from Fig. 4(a) in the main text and contains the following steps (each step is numbered on the figure for identification):

1. **Splitting**: we start from a pulse out of our OPO. This seed pulse is then split into a strong pump and a weak signal beams by means of a beam splitter.

2. **Pump Truncation:** we truncate the red part of the ultrashort pump pulse using an amplitude mask in the location of the spectral plane of a conventional pulse shaper apparatus (using a spatial light modulator). Alternatively, one can use a very sharp long-pass or short-pass filter. After this truncation the spectral shape of the pump is as follows:

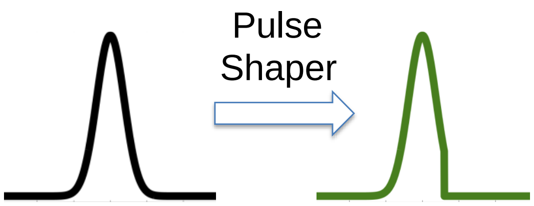

3. **Delay:** In a different beam path we insert a delay line for the signal beam in order to compensate for the delay between then pump and signal beams inserted by the pulse shaper and other optics elements along the setup.

4. **Signal Truncation:** we truncate most of the signal pulse using a very sharp long-pass filter.

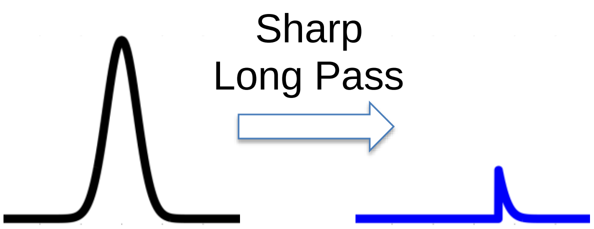

As a result, the signal and the pump are now spectrally discriminable.

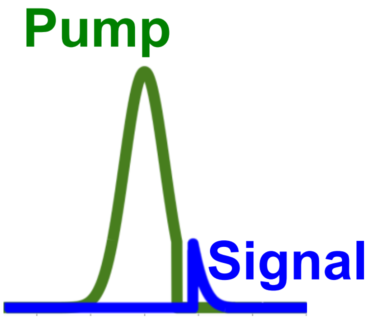

5. **Coupling into the sample**: the signal beam is coupled to the outer waveguide #1 whereas the pump beam is coupled to the middle waveguide (#2). There, because of the relatively high intensity of the pump beam, the refractive index of the middle waveguide is modulated due to the Kerr effect $n_{middle} = n + n_{Kerr} I_{pump}$. As a result the effective coupling between waveguides 1 and 3 is modulated and this is observed in Fig 4 when the pump and the signal are synchronized (i.e. they travel through the device at the same time).

6. **Elimination of the pump spectral part and Detection**: Only the output from the outer waveguide is collected in the far-field. The elimination of the background pump spectral part is achieved by using a sharp long pass filter. In our case we have used the *RazorEdge ultrasteep* filters from Semrock (1319 nm, identical to the one used in step 4). Those filters have above an OD 6 change within 3 nm bandwidth, meaning that the filter reduces the pump by 6 orders of magnitude while maintaining the signal beam. Only the output from the outer waveguide is collected in the far-field.

   Note that we ensure that, while weak, the signal beam is still significantly stronger than the four wave mixing generated in the middle waveguide.

7. **Synchronization**: the spectrally distinct pump and probe are combined into a BBO crystal. When synchronized, the two beams mix through the second order nonlinearity ($\chi^{(2)}$) of the crystal and generate a sum frequency (see Supplementary Figure 9).

Supplementary References:

1. Boyraz, Ö., Koonath, P., Raghunathan, V., Jalali, B. All optical switching and continuum generation in silicon waveguides. *Opt. Express* **12**, 4094–4102 (2004).
2. Lin, Q., Painter, O. J. & Agrawal, G. P. Nonlinear optical phenomena in silicon waveguides: modeling and applications. Opt. Express 15, 16604–16644 (2007).
3. Dulkeith, E., Xia, F., Schares, L., Green, W. M. J. & Vlasov, Y. A. Group index and group velocity dispersion in silicon-on-insulator photonic wires. Opt. Express 14, 3853–3863 (2006).
4. Reed, G. et al. in Silicon Photonics for Telecommunications and Biomedicine Ch. 1 p. 26 (CRC Press, 2011).
5. Liu, A. et al. High-speed optical modulation based on carrier depletion in a silicon waveguide. Opt. Express 15, 660–668 (2007).
6. Green, W. M., Rooks, M. J., Sekaric, L. & Vlasov, Y. A. Ultra-compact, low RF power, 10 Gb/s silicon Mach-Zehnder modulator. Opt. Express 15, 17106–17113 (2007).
7. Manipatruni, S., Xu, Q., Schmidt, B., Shakya, J. & Lipson, M. High Speed Carrier Injection 18 Gb/s Silicon Micro-ring Electro-optic Modulator. in LEOS 2007 - IEEE Lasers and Electro-Optics Society Annual Meeting 537–538 (IEEE, 2007). doi:10.1109/LEOS.2007.4382517
8. Xu, Q., Manipatruni, S., Schmidt, B., Shakya, J. & Lipson, M. 12.5 Gbit/s carrier-injection-based silicon micro-ring silicon modulators. Opt. Express 15, 430–436 (2007).
9. Watts, M. R., Trotter, D. C., Young, R. W. & Lentine, A. L. Ultralow power silicon microdisk modulators and switches. in 4–6 (IEEE, 2008). doi:10.1109/GROUP4.2008.4638077
10. Zheng, X. et al. Ultra-low-energy all-CMOS modulator integrated with driver. Opt. Express 18, 3059 (2010).
